# On Rheumatism in the Heart, Eyes, &c.

**Published:** 1816-09

**Authors:** James Jackson

**Affiliations:** Professor of the Theory and Practice of Physic in Harvard University.


*For the London Medical and Physical Journal.*

*On Rheumatism in the Heart, Eyes, &c.;* by JAMES JACKSON, M.D. Professor of the Theory and Practice of Physic in Harvard University.

THE occurrence of affections in the heart and lungs in persons affected with rheumatism, is neither new, nor very rare. Yet the history of such affections has been, within a few years, ascertained more definitively and precisely than at any former period. Dr. George Fordyce, in his third Dissertation on Fever, says, that, when bleeding is practised in acute rheumatism, a fatal metastasis to the head or breast is frequently produced. Although he might be in error as to the cause, his remark shows that the metastasis was well known to him. The remark has been more strongly impressed on my mind in consequence of a case, which occurred in St. Thomas's Hospital, about the time when I read it. This was the case of a young man under the care of Dr. Ainslie. The patient had acute rheumatism, and was bled copiously. Dr. Fordyce had two or three cases of acute rheumatism under his care at the same time, in which the affection was equally severe; and he did not direct bleeding. His patients recovered very well; but Dr. Ainslie's patient was affected with a severe disease of the heart and lungs, in consequence of which he had great dyspnœa, and was obliged to keep his body in an erect position. It was remarkable that the pulse could be felt in only one wrist, in which it was very strong and hard; and my impression was, that the pulse had been felt in both wrists at the commencement of the disease. Repeated bleedings and blistering were tried without relief, and the patient at length sunk, after excruciating sufferings.

Instances of a similar affection of the heart must, doubtless, have occurred at all times; and there may be found other occasional notices of them, or references to them, besides that of the philosophical observer above referred to. That, however, a real inflammation of the heart and lungs is established in such cases, has been unequivocally demonstrated only by late observers. In three different periodical works, of recent date, we find papers on this subject. The first is by Mr. Dundas, serjeant-surgeon to the king, published in the Medico-Chirurgical Transactions, vol. i. in 1809. The second is by Charles W. Wells, M.D. physician to St. Thomas's Hospital, and published in 1812, in the 3d vol. of the Transactions of a Society for the Improvement of Medical and Chirurgical Knowledge. In this paper, Dr.

Dr. Wells gives to the late Dr. David Pitcairn the credit of first remarking "that persons subject to rheumatism are attacked more frequently than others with symptoms of an organic disease of the heart."

The third paper is in the "Journal Général de Médecine, &c. redigé par Sedillot, No. CCXXII, Tome LII," published in February 1815, and is written by Mr. Matthey, D. M. P. of Geneva.

Most of the cases related in these papers proved fatal, and several of them were examined after death. A few of them terminated more favourably; but some of the patients continued for a long time to manifest a disorder in the functions of the heart; and others continued to suffer from this cause at the period when their cases were published. The symptoms were those common to carditis and pericarditis; and with them were sometimes combined from the beginning symptoms, which show that the lungs also were affected with inflammation. The examinations after death shew that inflammation had proceeded to the adhesive stage in the pericardium, and often both in the serous and in the cellular membrane of the lungs. The lungs were also disordered in consequence of the irregularity of the circulation, as well as from the direct effect of inflammation on them.

For many interesting and important details the reader is referred to the very valuable papers above described. They will abundantly repay him for a careful perusal. He will find in all of them evidence of unequivocal inflammation of the heart taking place in subjects, who at the moment, or shortly before, were affected with rheumatism. He will find that this inflammation rarely, if ever, passes beyond the adhesive stage; but that, so far, it exhibits the characteristics of common inflammation. But whether this affection is specifically of the same kind as that affecting the large joints in rheumatism may admit a doubt. That however the heart and also other parts, not commonly subject to this disease may be truly affected by it, will appear from the cases now to be related.

Case I.—In July 1812, I was called to attend Miss C. a young lady, then about twenty years of age. She underwent the acute rheumatism in a very severe form, so that I attended her closely for about six or seven weeks, and occasionally for some weeks afterwards. The disease attacked most of the large joints, also the neck and loins, and affected most of these parts three distinct times. In each of the circuits which it thus made, it included the heart and the muscles of the thorax. When these parts were affected the disease diminished and gradually subsided in the joints last seized, and, as the heart became relieved, the disease again seized on some of the joints. But the limbs were not entirely relieved during the affection of the heart, any more than one joint was entirely relieved during the affection of another joint. The symptoms, which marked the affection of the heart and thoracic muscles, were pain and distress in the region of the heart and across the breast, difficulty of breathing and even of speaking, violent palpitation, great tendency to syncope especially on motion and irregularity of the pulses. These were mostly very frequent, but at one time were very slow; they often intermitted and were very irregular. This patient was visited several times by my late respected colleague John Warren, M.D. At one period we were both in momentary expectation of the termination of life in the young lady, in consequence of the great difficulty with which the heart performed its functions. For moments it would pause and seemed to threaten to stop for ever.

The patient was of a slender form and feeble constitution, and the first attack on the heart was not extremely severe. She was not bled therefore at that period. And although the subsequent attacks were much more severe, and especially the second, she was then too much reduced to leave a question on the expediency of that evacuation. The remedies were vesication and opium, from both of which she derived great relief during the affections of the heart; also antimonials, submuriate of quicksilver, and, in the later stages, cinchona and other tonics. The patient survived the disease contrary to our expectations, and although her convalescence was slow, yet ultimately she recovered her health entirely. She was married shortly afterwards, and in the following autumn was safely delivered of a healthy child.

From the first attack on the heart, this organ continued unusually irritable during the remaining period of the disease and of the convalescence; just as those joints, which suffered severely were not, during the same period, entirely relieved from lameness. While the heart was much affected the patient found it necessary to have her head and shoulders raised very high, and even at times to have the trunk in an erect position; at these periods also the least motion and even speaking were very distressing to her; but at other times she could lie down and submit to some variation of posture.

Case II. In the autumn of 1814, Mrs. L. an English lady, came under my care on account of acute rheumatism, with which she was severely affected for several weeks. The disease affected her neck and the large joints of the limbs, likewise the hands and feet. It began in the large joints and its character was unequivocal. During its continuance it changed from from limb to limb as usual, and the different parts were affected a second time. In its circuit each time it attacked the eyes. The tunica conjunctiva of both eyes became suddenly and violently inflamed, and continued to be so for three or four days; then they mended suddenly, but at that moment the nose became diseased. The disease appeared here to affect the mucous membrane. In the course of the disease the parts about the prœcordia were severely affected, and respiration was very painful. This is not rare in acute rheumatism.

In this case I could not doubt that the ophthalmia was rheumatic, and I had before seen some appearances of the same kind, but which had left only a slight impression on my mind. The following case which happened fifteen months afterwards was sufficient of itself to decide the question, whether the eyes could be affected with rheumatism.

Case III.—This is the case of Mr. P. a gentleman who was about twenty years of age and of a sanguine temperament. He had generally enjoyed good health.

On the 13th of January (1815,) he called on me with the tunica conjunctiva of his right eye slightly inflamed. On moving the eye he complained of some pain, which appeared to be in the proper muscles of that organ. I directed some leeches and a saturnine lotion. These remedies were employed that evening, but notwithstanding them the inflammation increased very greatly during night, and the pain was extremely violent. Early the next morning I found the tunica conjunctiva very much swollen and very red, the cornea opake, and the powers of vision entirely lost. The skin was hot, and the pulses hard and accelerated. I bled him freely from the arm twice on that day, purged him and blistered his neck. The pain had ceased at evening, but returned with great severity during the night. For five days the inflammation continued with great fury,—during which time he lost by general and local bleeding more than seven pounds of blood, was purged abundantly, blistered freely, kept in the dark and on the lowest diet. During this time the vessels of the conjunctiva were cut off by Dr. J. C. Warren, twice, so as to arrest the disease in the cornea. As the cornea mended, the aqueous humour became turbid. On the sixth day the inflammation was yielding, and on the seventh its violence had quite subsided. The cornea and aqueous humour very soon recovered their usual transparency; but the powers of vision were scarcely mended, owing I presume to an affection of the nerve.

It should be remarked that this inflammation was very rapid pid in its changes, affecting in succession parts of different structure in the eye, and that it was extremely violent in degree, resisting the effects of remedies of great power. The pulses at the same time were very hard. Although the bleedings were large and produced deliquium in every instance, yet the pulses scarcely yielded to them until the fifth day on which they were practised, nor then perfectly. This was the sixth day of the disease. On the evening of the seventh day the nature of the affection was manifested for the first time by a transference of it to the right knee. This joint was then attacked with pain and soon after by swelling evidently rheumatic, and at the same time the pain left the eye entirely.

From this period the disease manifested the common symptoms of acute rheumatism, affecting the large joints of the lower extremities for the most part, and occasionally the neck and loins. It continued to renew its attacks with severity for three months, although there was in this time one interval of a month, in which the disease appeared to be subsiding. The eye had two attacks during these three months, the disease leaving the limbs and returning to them again as the eye recovered. The second attack was short but severe, though less so than the first. The third was less severe, but longer. In each of them the blindness was perfect; and in each the disease affected first the tunica conjunctiva, then the cornea, then the aqueous humour. In one instance the crystalline humour was opake for a day or two. The changes of structure took place suddenly and subsided suddenly, as happens in respect to the swelling of the joints in this disease.

When the eye was attacked the second time, blood was drawn from the arm, and in each attack leeches were applied liberally. Vesication was employed almost constantly about the head and on the limbs. Antimonials were given very freely, both before and after the real nature of the disease was manifested;—opium was exhibited p. r. n.—and in the course of the disease cinchona was tried very fully. The strength of the patient was less reduced than might be expected from the evacuations, and never so much prostrated as is common in acute rheumatism. Great care was taken in the use both of food and medicine not to reduce the stomach to the irritable state so common in this disease; and although the appetite was lost in the early stage that organ never lost its tone, and nothing like dyspepsia took place at any period.

The joints of the upper extremities were almost exempted from disease; but those of the lower extremities (the knee and and ancles) were so severely affected that the patient was unable to walk a step from the first attack until about the 1st of May. During perhaps the largest part of this time he had very little pain when at rest.

The power of vision in the rheumatic eye was never restored perfectly; but the precise state of it was not ascertained until April, when for the first time the light was admitted freely into the patient's apartment. He then found that there was frequently occurring an appearance of something like the body of a spider, with two wings, passing over his eye at the upper part of the pupil. As there was not any mark of injury in the cornea, nor of change in the humours, this appearance was attributed to an affection of the retina. This opinion was confirmed by the consideration that the power of vision was restored very slowly after each attack, although the opacities had subsided suddenly and rapidly; and by the statement of the patient that he had frequently noticed this appearance before he mentioned it and while the room was darkened, but that he had thought it was owing to the defect of light in the room.

In the latter part of April and in May electrical shocks were applied to the head three times a day. Also mercurials were used moderately—i. e. Subm. Hyd. gr. i. every night. The eye recovered partially, but not entirely.

Mr. P.'s mother has been very often, and at times severely, affected with rheumatism. Two gentlemen, cousins of hers, have been nearly crippled for many years with the same disease, and their eyes have been so much affected as to have rendered them nearly blind. This affection of the eyes has evidently been connected with the disease in the limbs.

When the rheumatic character of the ophthalmia in this case was first developed, and I expressed my opinion respecting it to Dr. Warren, to whom I stated the recent change at his subsequent visit, he remarked at once that he had not any doubt on the subject; for that he had previously seen more than one case of a similar nature in his own practice. An account of these cases he is about to publish, with some others of diseases in the eye.

Case IV.—At the moment while preparing this paper for the press, (March 1816) I have under my care a lady nearly seventy years of age, who has acute rheumatism, and in whom during the last week, the tunica conjunctiva of both eyes has been inflamed. The inflammation of the eyes in her case came on rather slowly, has not been attended with severe pain, though the swelling has been considerable, and has not been accompanied by any marked alleviation of the disease in the limbs. It is true, however, in this case that the limbs have suffered less during the ophthalmia than before.

CASE V.—To the foregoing cases I may add one more, of a kind which I apprehend has been more noticed than either of the others. During the last autumn dysentery was more common in this place than usual. About the beginning of October, I attended Mr. H. under this disease. The symptoms yielded at once to remedies, but did not quite subside for eight or ten days. About this period the patient began to complain of stiffness in moving his limbs, particularly his thighs. On the second day this affection was evidently rheumatic, and the dysentery ceased at this moment. The rheumatism diminished after two or three days, under the use of common remedies, but it did not entirely subside. There now occurred symptoms of disordered stomach, induced apparently by imprudence in diet. An emetic was administered which operated very violently, and from this time the patient recovered rapidly.

Cases of this kind have been noticed by Dr. Ferriar and others, and I should not have thought this worthy of publication, except in connection with what follows. A short time since I had a patient with pneumonia, in whom as the pneumonia subsided there took place rheumatism in the muscles of the neck, with great pain in the head. In this stage of the disease a physician, who practises in the western part of the state of New York, and who was related to the patient, happened to be in town. He saw the patient with me, and, in conversation respecting the case, he stated that during the last autumn dysentery had prevailed in his neighbourhood, and that in a very considerable proportion of his patients it had been followed by rheumatism; the dysentery subsiding as the rheumatism supervened. This is the first instance, so far as I know, in which such a metastasis has characterized an epidemic. The gentleman above referred to was one of liberal education, and appeared to me to be entitled to full credit.

I trust that the facts stated in this paper will not be destitute of utility; but I will leave to the reader the task of making inferences from them.—*New-England Journal.*

